# Impact of coexisting obesity and malnutrition on the clinical outcomes of acute ischemic stroke patients treated with intravenous thrombolysis: a cohort-based study

**DOI:** 10.3389/fnut.2026.1864875

**Published:** 2026-07-31

**Authors:** Huicong Niu, Xuechun Wu, Yi Chen, Ziwen Zhu, Xueyu Mao, Shiliang Xia, Min Chu

**Affiliations:** 1 Department of Neurology, Minhang Hospital, Fudan University, Shanghai, China; 2 Department of Geriatrics, Shanghai Geriatric Medical Center, Shanghai, China

**Keywords:** acute ischemic stroke, intravenous thrombolysis, obesity, prognosis, the double burden of malnutrition

## Abstract

**Background:**

The simultaneous presence of obesity and malnutrition, termed the double burden of malnutrition (DBM), has become a growing public health challenge. However, little is known about how the DBM influences patients with acute ischemic stroke (AIS) who receive intravenous thrombolysis (IVT). We aimed to investigate the associations between obesity and nutritional status and clinical characteristics and clinical outcomes among AIS patients receiving IVT.

**Methods:**

We conducted a single-center, prospective cohort study at a Shanghai hospital, including AIS patients who received alteplase IVT therapy from January 2018 to December 2022. Patients were categorized into four distinct subgroups based on body mass index-based obesity and nutritional status, as assessed using the Controlling Nutritional Status score: (1) nourished non-obese; (2) malnourished non-obese; (3) nourished obese; and (4) malnourished obese. The primary endpoints of the study were poor outcomes and all-cause mortality within 3 months, while the secondary outcomes were early neurological deterioration (END) and hemorrhagic transformation (HT).

**Results:**

The overall stroke cohort consisted of 6,892 AIS admissions, of which 366 patients receiving IVT were included in this study. The median age was 68 ± 12 years, and 239 subjects (65.3%) were men. A total of 52.7% of patients were malnourished, and greater malnutrition severity independently predicted worse clinical outcomes after thrombolysis. During the follow-up period, the malnourished obese group had the highest rates of poor outcomes and END, followed by the malnourished non-obese, nourished obese, and nourished non-obese groups (*p* < 0.05). After adjusting for confounding factors in a multivariate logistic regression analysis, the malnourished obese group showed significantly elevated risks of poor outcomes (odds ratio [OR], 2.850 [1.061–7.650]; *p* = 0.038) and END (OR, 19.158 [2.324–157.935]; *p* = 0.006), using the nourished non-obese group as the reference group.

**Conclusion:**

Malnutrition affected more than half of AIS patients undergoing IVT, including those with obesity. Patients with malnutrition were associated with worse clinical outcomes, especially those with moderate-to-severe malnutrition. Notably, patients with concurrent obesity and malnutrition exhibited the most unfavorable 3-month clinical profiles across all four subgroups.

## Introduction

Stroke is a leading cause of adult mortality and disability worldwide, with ischemic stroke accounting for 41–87% of all strokes, and the global burden of disability caused by stroke has increased in recent years (1, 2). Intravenous thrombolysis (IVT) is one of the most effective treatments for acute ischemic stroke (AIS) (3, 4). However, approximately half of the patients with AIS fail to achieve favorable outcomes, even after receiving IVT (3). Timely and accurate evaluation of patients’ conditions can help neurologists identify reliable predictors of IVT clinical outcomes and adjust therapeutic regimens to improve patient prognosis.

Obese individuals, typically defined by body mass index (BMI), are highly heterogeneous, with a wide metabolic spectrum. In metabolically unhealthy individuals, obesity often coexists with multiple comorbidities, such as diabetes, hypertension, dyslipidemia, and sleep apnea, which may contribute to deleterious systemic and stroke effects (5). On the other hand, some individuals with obesity remain metabolically healthy, suggesting that obesity alone may not be an independent risk factor for ischemic stroke (6). There remains a strong need to better understand the varying predispositions toward the risk of stroke complications (7).

The double burden of malnutrition (DBM)—the simultaneous occurrence of obesity and malnutrition—has emerged as a prevalent global public health challenge (8–10). This concept challenges the traditional view that obesity equates to adequate nutrition. The Controlling Nutritional Status (CONUT) score is a surrogate marker of nutritional status, which is crucial for physiological function and has been evidenced to be a prognostic predictor of clinical outcomes in multiple diseases, particularly AIS (11–13). Previous studies have demonstrated that malnutrition, denoted by a higher CONUT score, is associated with early neurologic deterioration (END) and a higher risk of mortality in AIS patients after IVT, which might be mediated by increasing erythrocyte aggregation, reducing antioxidant effects, and promoting endothelial injury, among other pathways (13, 14). Malnutrition may further exacerbate stroke-related adverse effects in patients with chronic obesity. At present, no research has explored the joint impact of obesity and malnutrition on the prognosis of AIS patients receiving IVT. Previous studies were limited to investigating the separate effects of either obesity (15, 16) or nutrition (13, 17, 18) on AIS patients. In this study, we aimed to analyze the associations between the simultaneous manifestation of obesity and malnutrition and the prognosis of AIS patients who underwent IVT.

## Methods

### Subjects and study design

**Figure 1:**
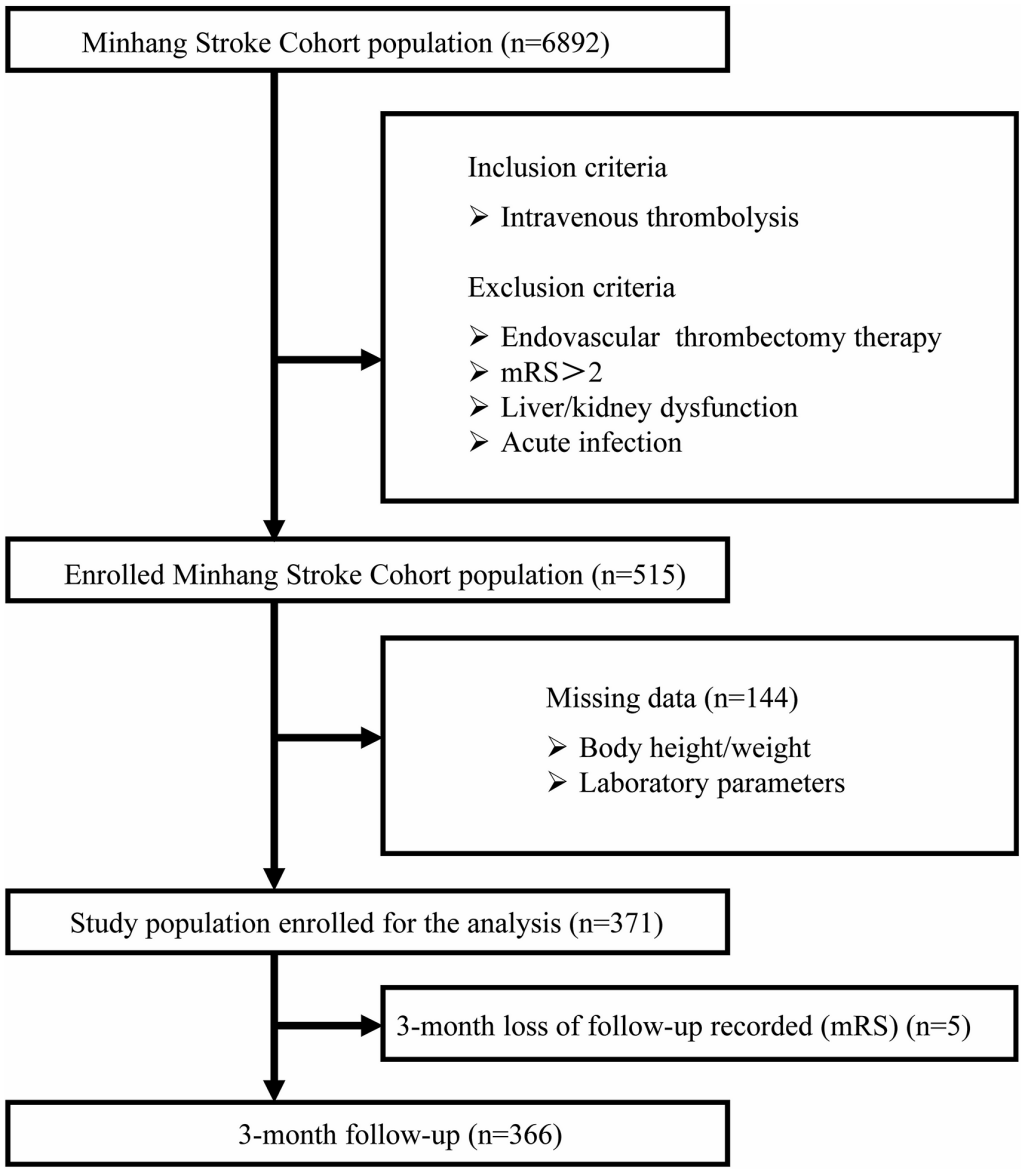
Flowchart illustrating the screening and inclusion process of study participants.

The present study used data from the Minhang Stroke Prospective Observational Cohort, which included a total of 6,892 patients with ischemic stroke treated at a Shanghai hospital from January 2018 to December 2022. Consecutive participants were eligible for this IVT-focused analysis if they met three inclusion criteria: (1) an AIS diagnosis verified via cranial computed tomography (CT) or magnetic resonance imaging (MRI); (2) alteplase IVT administered within 4.5 h of stroke symptom onset; and (3) no history of endovascular mechanical thrombectomy. Patients were further excluded if they met one of the following criteria: a prestroke modified Rankin Scale (mRS) score of >2 (*n* = 10); a medical history of liver/kidney dysfunction or acute infection within 2 weeks before admission (*n* = 33); missing measurements of weight, height, or key laboratory indicators, including lymphocyte count, albumin level, and total cholesterol level (*n* = 144); or incomplete 3-month follow-up records (*n* = 5). Ultimately, 366 eligible participants formed the study sample, as illustrated in the participant screening flowchart (Figure 1). Ethical clearance for this cohort study was granted by the Institutional Ethics Committee of Minhang Hospital, Fudan University, and all study procedures followed the ethical principles outlined in the Declaration of Helsinki.

Clinical and laboratory data were collected from all patients, and patients were stratified into four groups based on their nutritional and obesity status: (1) nourished non-obese; (2) malnourished non-obese; (3) nourished obese; and (4) malnourished obese. Nutritional status stratification relied primarily on the externally validated CONUT scoring tool, calculated by summing three subscores derived from serum albumin, total cholesterol, and peripheral lymphocyte counts. Score ranges of 0–1, 2–4, 5–8, and 9–12 corresponded to normal nutrition and mild, moderate, and severe malnutrition, respectively (Supplementary Table S1) (19). Two additional nutritional assessment tools (the Geriatric Nutritional Risk Index [GNRI] and the Prognostic Nutritional Index [PNI]) were adopted for supplementary analysis. The GNRI was calculated using the following formula: 1.489 × serum albumin [g/L] + 41.7 × present body weight [kg]/ideal body weight [kg] (20). The ideal body weight was calculated according to the Lorentz equation: height (cm) − 100 − [(height (cm) − 150)/4] for men and height (cm) − 100 − [(height (cm) − 150)/2.5] for women (11). All patients were categorized into the following four groups according to their GNRI scores: no risk (>98), mild risk (92–98), moderate risk (82 to <92), and severe risk (<82) groups (20). The PNI was calculated as follows: serum albumin concentration (g/L) + 5 × total lymphocyte count (10^9^/L). PNI scores of >38, 35 to 38, and <35 were defined as indicating normal, moderate, and severe malnutrition status, respectively (21) (Supplementary Table S1). The CONUT score was used for subsequent analysis because of its superior predictive power compared with the GNRI and PNI (7). For these analyses, moderate and severe malnutrition were combined into a single category representing moderate-to-severe malnutrition risk.

Consistent with the World Health Organization BMI thresholds for Asian populations, obesity was defined as a BMI of ≥ 27.5 kg/m^2^ (22, 23). Blood samples were collected within 24 h of admission and analyzed in the laboratory at Minhang Hospital. All biochemical indicators were tested using the Cobas 800 auto-analyzer from Roche Diagnostics (Indianapolis, IN, United States). Renal dysfunction was defined as a serum creatinine level of >110 μmol/L or an estimated glomerular filtration rate (eGFR) of < 60 mL/1.73 m^2^. The abbreviated equation derived from the Modification of Diet in Renal Disease research was used to calculate eGFR: eGFR = 186 × [Scr]^−1^·^154^ × [age]^−0^·^203^ × (0.742 for women) × 1.233 (where Scr represents serum creatinine and 1.233 is the adjustment coefficient for the Chinese population) (24). Liver function was considered abnormal if aspartate aminotransferase (AST) was > 66 U/L or alanine transaminase (ALT) was > 40 U/L.

### Clinical assessment and outcome measurement

The severity of a patient’s neurological deficits was assessed at admission using the National Institutes of Health Stroke Scale (NIHSS), which is a gold-standard, quantitative scale that comprehensively assesses consciousness, visual fields, facial movement, limb motor function, sensory function, language, and ataxia. The mRS was used to assess patients’ functional status. Clinical information on patients’ outcomes after discharge was obtained prospectively by examiners blinded to the clinical parameters and biomarker levels during routine clinic visits or via telephone interviews with patients or their caregivers. Details about deceased patients were gathered from their relatives and validated using death certificates obtained from local civil registries or the corresponding treatment hospitals. The primary endpoints were poor outcomes (mRS score of 3–6) and all-cause mortality at 3 months after the qualifying event. The secondary endpoints were END, defined as neurological deterioration with a worsening in the NIHSS of 4 or more points, and hemorrhagic transformation (HT), defined as any visible hemorrhage on a brain CT after IVT during hospitalization.

### Statistical analysis

SPSS version 26.0 (IBM, Armonk, NY, United States) was used for all statistical analyses. Categorical variables were analyzed using chi-square tests, while a one-way ANOVA was used for between-group comparisons of continuous variables. Three discriminative metrics, including the c-statistic, continuous net reclassification improvement (NRI), and integrated discrimination improvement (IDI), were computed to assess the predictive ability of the three malnutrition assessment tools for predicting 3-month all-cause mortality and poor outcomes. Logistic regression models were further used to evaluate the independent association between combined nutritional status and obesity and clinical outcomes during the follow-up period, after adjusting for age, admission NIHSS score, male sex, door-to-needle time (DNT), smoking status, alcohol consumption, stroke subtype, diabetes, prior stroke, hypertension, atrial fibrillation, coronary heart disease, and statin use. A secondary analysis concerning the independence of malnutrition severity on clinical outcomes was adjusted for obesity, age, admission NIHSS score, male sex, DNT, smoking status, alcohol consumption, stroke subtype, diabetes, prior stroke, hypertension, atrial fibrillation, coronary heart disease, and statin use. Statistical significance was set at a *p-*value of ≤0.05.

## Results

### Baseline characteristics

**Figure 2:**
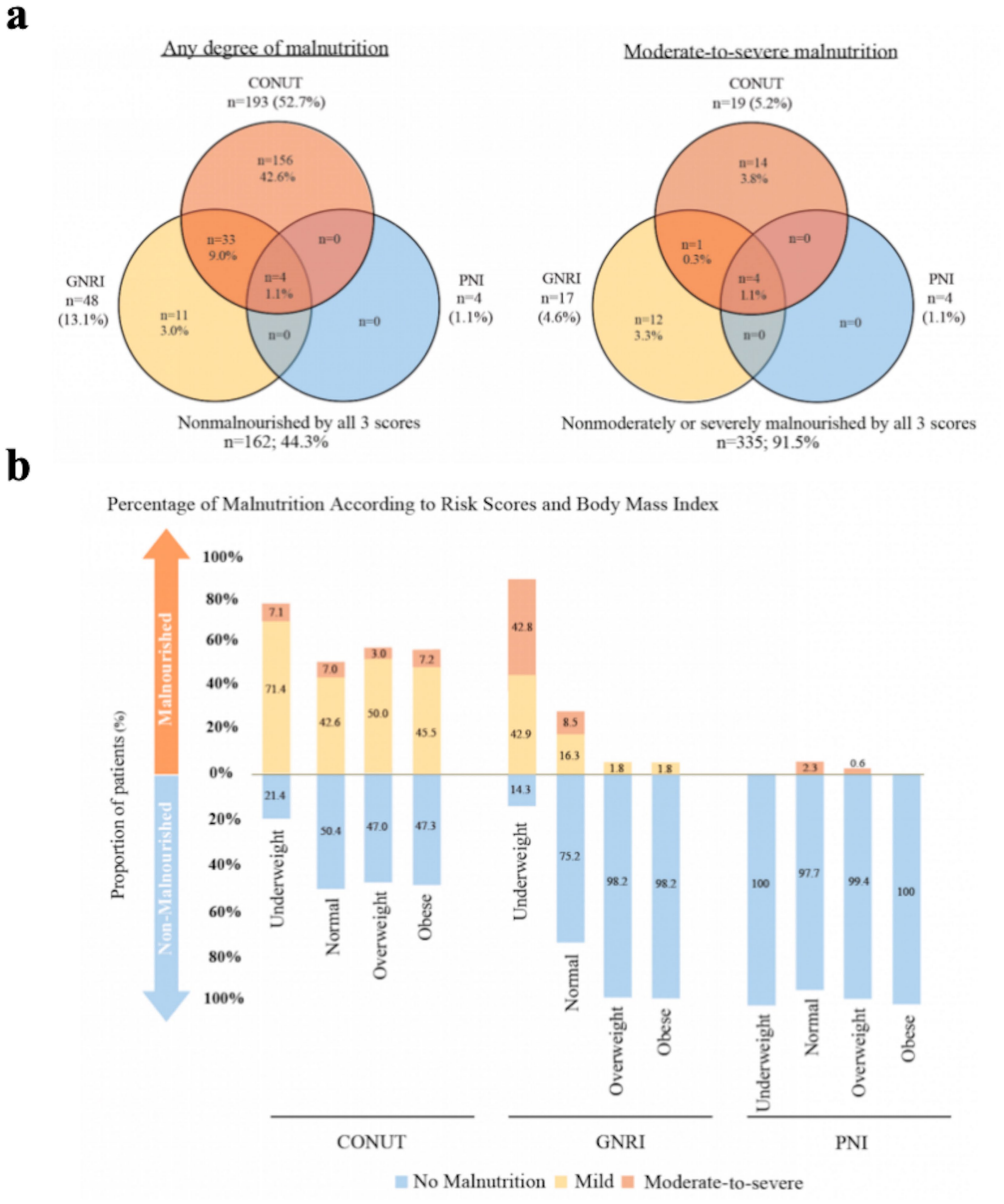
Prevalence of malnutrition evaluated via three distinct nutritional scoring tools. **(a)** Venn diagrams depicting overlapping malnutrition cases identified by the three scoring systems. The overlapping regions of the circles indicate patients concurrently diagnosed with malnutrition by multiple indices (left: all grades of malnutrition; right: moderate-to-severe malnutrition). **(b)** Proportions of patients with different malnutrition grades stratified by BMI and the three nutritional assessment scores. CONUT, Controlling Nutritional Status; GNRI, Geriatric Nutritional Risk Index; PNI, Prognostic Nutritional Index; BMI, body mass index.

The Minhang Stroke Cohort consisted of 6,892 patients with ischemic stroke, and 366 IVT-treated subjects were selected as the analytical sample of this study (mean age: 68 ± 12 years, 65.3% male patients; Table 1). Baseline comparisons between this study sample and the full cohort are presented in Supplementary Table S2. The percentage of patients with malnutrition varied according to the assessment tool, ranging from 1.1% using the PNI to 13.1% using the GNRI and 52.7% using the CONUT score (Figure 2a and Supplementary Table S3). Although all three malnutrition indices were correlated with each other, only 1.1% of patients were classified as malnourished (at any degree) by all three assessment tools, and 44.3% were classified as not malnourished by any of the three tools (Figure 2a). The CONUT score and the PNI outperformed the GNRI in predicting poor outcomes and all-cause mortality, as demonstrated by their discriminative index values. However, the CONUT score showed greater sensitivity than the PNI for both outcomes (Supplementary Table S4). Therefore, the CONUT score was used to evaluate the malnutrition status in the subsequent analyses. In the study population, 15.0% of the patients were obese, 45.9% were overweight, 35.2% were of normal weight, and 3.8% were underweight. The highest prevalence of malnutrition was observed among underweight patients (BMI < 18.5 kg/m^2^) according to both the CONUT (78.5%) and GNRI (85.7%) scores. Notably, a substantial proportion of obese patients were also classified as malnourished according to the CONUT score (Figure 2b).

**Table 1:** Baseline demographics of the study population.

Parameter	Overall	Nourished non-obese (*n* = 147)	Malnourished Non-obese (*n* = 164)	Nourished obese (*n* = 26)	Malnourished obese (*n* = 29)	*p*-value
Age (years)	68 ± 12	66 ± 12	70 ± 11	62 ± 15	71 ± 15	0.001
Sex—male patients, *n (%)*	239 (65.3%)	93 (68.3%)	113 (68.9%)	17 (65.4%)	16 (55.2%)	0.479
CONUT	1.8 ± 1.5	0.6 ± 0.5	3.0 ± 1.1	0.2 ± 0.4	2.8 ± 1.1	<0.001
BMI	24.1 ± 3.4	23.1 ± 2.2	23.0 ± 2.6	30.0 ± 2.3	29.3 ± 2.0	<0.001
Severity of malnutrition based on CONUT score						
None	173 (47.3%)	147 (100%)	0	26 (100%)	0	NA
Mild	174 (47.5%)	0	149 (90.9%)	0	25 (86.2%)	
Moderate-to-severe	19 (5.2%)	0	15 (9.1%)	0	4 (13.8%)	
Weight group						
Underweight (BMI < 18.5)	14 (3.8%)	3 (2.0%)	11 (6.7%)	0	0	NA
Normal (BMI 18.5–22.9)	129 (35.2%)	65 (44.2%)	64 (39.0%)	0	0	
Overweight (BMI 23–27.4)	168 (45.9%)	79 (53.7%)	89 (54.3%)	0	0	
Obese (BMI ≥ 27.5)	55 (15.0%)	0	0	26 (100%)	29 (100%)	
Smoking status, *n* (%)	114 (31.1%)	46 (31.3%)	47 (28.7%)	13 (50.0%)	8 (27.6%)	0.176
Alcohol consumption, *n* (%)	52 (14.2%)	21 (15.0%)	21 (12.8%)	5 (19.2%)	4 (13.8%)	0.796
Medical history, *n* (%)						
Hypertension	217 (59.3%)	83 (56.5%)	101 (61.6%)	13 (50.0%)	20 (69.0%)	0.412
Diabetes mellitus	108 (29.5%)	35 (23.8%)	50 (30.5%)	12 (46.2%)	11 (37.9%)	0.077
Stroke	45 (12.3%)	18 (12.2%)	23 (14.0%)	1 (3.8%)	3 (10.3%)	0.585
Hyperlipidemia	197 (53.8%)	107 (72.8%)	56 (34.1%)	25 (96.2%)	9 (31.0%)	<0.001
Coronary artery disease	25 (6.8%)	7 (4.8%)	14 (8.5%)	2 (7.7%)	2 (6.9%)	0.584
Atrial fibrillation	38 (10.4%)	9 (6.1%)	19 (11.6%)	0	10 (34.5%)	<0.001

CONUT, Controlling Nutritional Status; BMI, body mass index.

The malnourished non-obese group was the most prevalent (44.8%), followed by the nourished non-obese (40.2%), the malnourished obese (7.9%), and the nourished obese (7.1%) groups. Malnourished patients, whether obese or not, were generally older than the participants in the nourished groups. Higher rates of hyperlipidemia were observed in the nourished groups, whereas atrial fibrillation was less common in these two subgroups (Table 1). The four groups did not differ significantly in the occurrence of pulmonary infection, renal dysfunction, or liver dysfunction; nevertheless, the malnourished non-obese and obese groups tended to have the highest rates of pulmonary infection (Table 2).

**Table 2:** In-hospital management and complications of the study population.

Parameter	Overall	Nourished non-obese (*n* = 147)	Malnourished non-obese (*n* = 164)	Nourished obese (*n* = 26)	Malnourished obese (*n* = 29)	*P*-value
Admission NIHSS scores	6 ± 5	5 ± 4	6 ± 5	5 ± 3	9 ± 6	0.008
Systolic pressure (mmHg)	150 ± 21	148 ± 20	152 ± 22	149 ± 22	149 ± 21	0.360
Diastolic pressure (mmHg)	85 ± 13	84 ± 12	85 ± 13	91 ± 16	84 ± 13	0.107
Time from onset to hospitalization (h)	1.6 ± 0.9	1.6 ± 0.9	1.6 ± 0.9	1.3 ± 0.9	1.6 ± 0.9	0.373
DNT (min)	68 ± 35	68 ± 34	66 ± 36	54 ± 24	86 ± 39	0.008
Stroke subtype, n (%)						
LAA	199 (54.4%)	86 (58.5%)	86 (52.4%)	16 (61.5%)	11 (37.9%)	0.003
CE	58 (15.8%)	15 (10.2%)	31 (18.9%)	0	12 (41.4%)	
SAO	103 (28.1%)	43 (29.3%)	45 (27.4%)	9 (34.6%)	6 (20.7%)	
Others	6 (1.6%)	3 (2.0%)	2 (1.2%)	1 (3.8%)	0	
Laboratory tests						
Lymphocyte counts (10^9^/L)	1.6 ± 0.7	1.9 ± 0.5	1.1 ± 0.4	2.2 ± 0.6	1.4 ± 0.8	<0.001
Hemoglobin (g/L)	138.3 ± 17.2	141.6 ± 16.7	135.1 ± 16.1	145.0 ± 18.7	133.8 ± 20.1	0.002
TG (mmol/L)	1.9 ± 1.6	2.0 ± 1.2	1.6 ± 1.3	2.4 ± 0.9	2.2 ± 4.0	0.003
TC (mmol/L)	4.6 ± 1.2	5.1 ± 1.1	4.1 ± 1.0	5.4 ± 0.8	4.0 ± 1.2	<0.001
LDL (mmol/L)	3.0 ± 1.0	3.5 ± 0.9	2.6 ± 0.8	3.8 ± 0.8	2.4 ± 1.1	<0.001
HDL (mmol/L)	1.2 ± 0.3	1.2 ± 0.3	1.2 ± 0.4	1.1 ± 0.2	1.1 ± 0.3	0.299
Creatinine (μmol/L)	79.4 ± 22.1	78.8 ± 21.9	80.0 ± 23.6	75.5 ± 16.3	82.5 ± 19.2	0.664
Secondary outcomes						
Pulmonary infection	34 (9.3%)	8 (5.4%)	22 (13.4%)	1 (3.8%)	3 (10.3%)	0.076
Renal dysfunction	13 (3.6%)	4 (2.7%)	8 (4.9%)	0	1 (3.4%)	0.684
Hepatic Dysfunction	3 (0.8%)	1 (0.7%)	2 (1.2%)	0	0	1.0

NIHSS, National Institutes of Health Stroke Scale; mRS, modified Rankin Scale; LAA, large artery atherosclerosis; CE, cardioembolism; SAO, small-vessel occlusion; TG, triglyceride; TC, total cholesterol; LDL, low-density lipoprotein; HDL, high-density lipoprotein.

### Study outcomes

**Figure 3:**
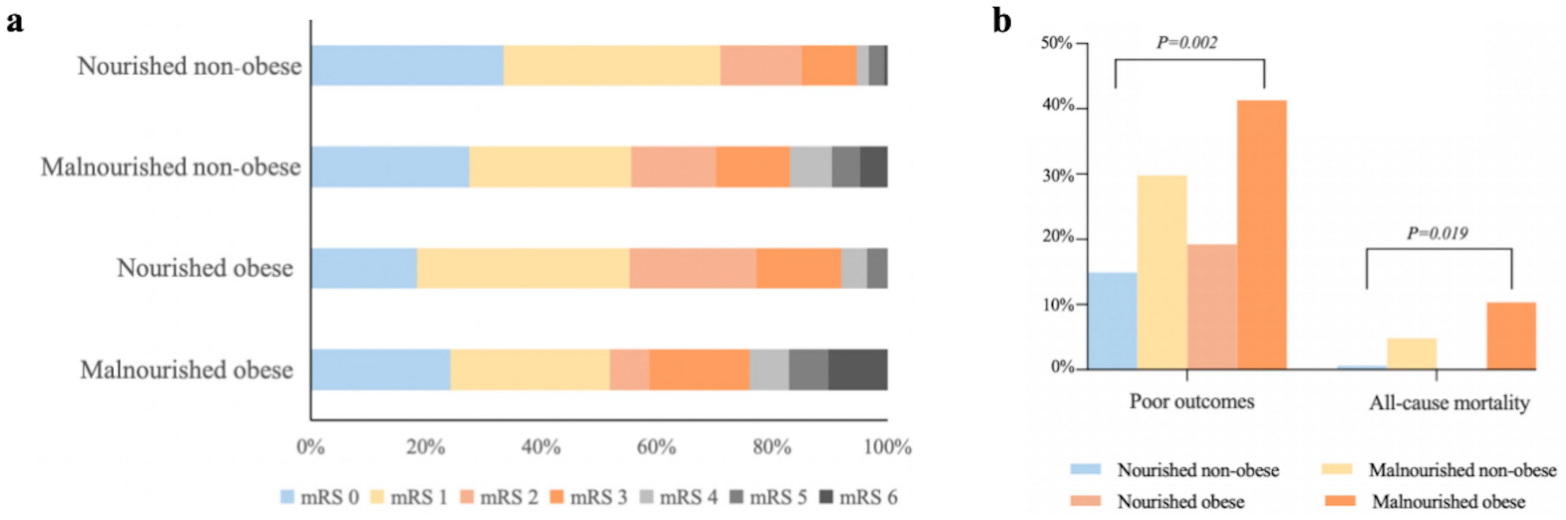
Distribution of 3-month stroke clinical outcomes across the four subgroups. **(a)** Intergroup comparison of 3-month mRS scores, with significant disparities observed among the groups. **(b)** Distribution of poor 3-month outcomes (mRS > 2, *p* = 0.002) and all-cause mortality across the four study subgroups (*p* = 0.019). mRS, Modified Rankin Scale.

During the follow-up period, 88 patients (24.0%) experienced poor outcomes (mRS > 2), 12 patients (3.2%) died, 15 patients (4.1%) experienced END, with 41 patients (11.2%) experiencing HT. The malnourished obese group had the highest rate of poor outcomes (41.3%), followed by the malnourished non-obese (29.8%), the nourished obese (19.2%), and the nourished non-obese groups (14.9%; *p* = 0.002; Table 3; Supplementary Table S5; Figure 3). An almost similar trend was observed for END, with the malnourished obese group showing the highest rate of END (13.8%), followed by the malnourished non-obese (4.9%), the nourished obese (3.8%), and the nourished non-obese groups (1.4%; *p* = 0.019; Supplementary Tables S5, S6 and Supplementary Figure S1). The differences in poor outcomes and END between the four groups were more strongly associated with nutritional status than with obesity, and the malnourished obese subgroup exhibited the least favorable 3-month clinical outcomes. In contrast, the nourished non-obese group had the lowest rates of poor outcomes and END throughout the follow-up period. In addition, malnourished patients had more unfavorable outcomes, with the malnourished obese group exhibiting the highest rates of all-cause mortality or HT (Table 3; Supplementary Tables S5, S6; Figure 3; Supplementary Figure S1).

**Table 3:** Adjusted poor outcomes and all-cause mortality according to nutrition and obesity status.

Clinical outcomes	Case (%)	OR (95% CI)	*P*-value
Poor outcomes	88 (24.0%)		
Nourished non-obese	22 (14.9%)	reference	
Malnourished non-obese	49 (29.8%)	1.947 (1.068–3.550)	0.030
Nourished obese	5 (19.2%)	1.398 (0.448–4.362)	0.564
Malnourished obese	12 (41.3%)	2.850 (1.061–7.650)	0.038
All-cause mortality	12 (3.2%)		
Nourished non-obese	1 (0.6%)	reference	
Malnourished non-obese	8 (4.8%)	6.021 (0.677–53.585)	0.107
Nourished obese	0 (0.0%)	NA	0.998
Malnourished obese	3 (10.3%)	11.502 (0.834–158.636)	0.068

Adjusted for age, admission NIHSS score, male sex, DNT, smoking status, alcohol consumption, stroke subtype, diabetes, prior stroke, hypertension, atrial fibrillation, coronary heart disease, and statin use. NIHSS, National Institutes of Health Stroke Scale; DNT, door-to-needle time.

Multivariable analysis showed that, using the nourished non-obese group as the reference group, the adjusted odds ratios (ORs) for poor outcomes were 2.850 (95% CI 1.061–7.650) and 1.947 (95% CI 1.068–3.550) for the malnourished obese group and the malnourished non-obese group, respectively; however, only a non-significant increase in poor outcomes was observed in the nourished obese group (OR,1.398; 95% CI 0.448–4.362; *p* = 0.564; Table 3). Similar results were observed for END. Moreover, the multivariable analysis of all-cause mortality did not reach significance, possibly due to the low event rate (Table 3). No significant difference in HT was observed using the nourished non-obese group as the reference, and the adjusted ORs for HT were 1.377 (95% CI 0.363–5.221) for the malnourished obese group, 1.966 (95% CI 0.874–4.429) for the malnourished non-obese group, and 0.673 (95% CI 0.078–5.788) for the nourished obese group (Supplementary Table S6).

In addition, malnutrition severity was significantly associated with poor outcomes after adjusting for confounders (mild malnutrition: OR, 1.786; 95% CI 1.011–3.154; *p* = 0.046; moderate-to-severe malnutrition: OR, 5.282; 95% CI 1.723–16.191; *p* = 0.004). The overall trends remained unchanged in the analysis of all-cause mortality, with malnutrition severity (mild malnutrition: OR, 6.240; 95% CI 0.696–55.964; *p* = 0.102; moderate-to-severe malnutrition: OR, 31.729; 95% CI 2.001–503.139; *p* = 0.014) being significantly associated with higher mortality. Similarly, moderate-to-severe malnutrition remained significantly associated with higher rates of END (OR, 17.368; 95% CI 1.661–181.572; *p* = 0.017) and HT (OR, 6.080; 95% CI 1.684–21.948; *p* = 0.006; Supplementary Table S7) after adjustment.

## Discussion

This study aimed to clarify the intricate interplay between obesity, nutritional status, and clinical outcomes in patients with AIS who underwent IVT. We found that (1) malnutrition was highly prevalent within the IVT cohort, and nearly half of the overweight or obese patients showed malnutrition according to the CONUT score; (2) malnutrition was associated with a poorer prognosis, and moderate-to-severe malnutrition independently predicted adverse clinical outcomes during the follow-up period; and (3) the concurrent presence of obesity and malnutrition were associated with poor clinical outcomes, and nourished non-obese individuals displayed the lowest burden of poor outcomes and END.

The high prevalence of malnutrition in AIS patients should be given due consideration. Over half of the AIS population in our study was malnourished (Figure 2), which was consistent with findings from the Third China National Stroke Registry (11). It is not surprising that malnutrition is quite prevalent among AIS patients because malnutrition is a growing global health concern and is not limited to socially deprived communities. Numerous studies have shown that malnutrition is significantly associated with poor prognosis in AIS, regardless of the malnutrition assessment tools used (14, 25). Similarly, this study also confirmed the positive relationship between malnutrition and poor outcomes, all-cause mortality, END, and HT in AIS patients who underwent IVT (Supplementary Table S7). The severity of malnutrition was correlated with accumulated atherosclerotic damage, a clinical pathway widely defined as the malnutrition–inflammation–atherosclerosis syndrome (26, 27). Additionally, post-stroke patients with malnutrition often experience electrolyte imbalances, anemia, infections, and gastrointestinal hemorrhage, leading to prolonged hospital stays and increased intensive care unit utilization (28). These findings highlight the importance of systematic nutritional screening in clinical practice, and individualized nutritional support should be prioritized.

### Nutritionally unhealthy obesity—a distinct obesity phenotype

The most crucial finding of our study focused on characterizing a new malnourished obese phenotype. We demonstrated that malnourished obese individuals were primarily women (Table 2). Indeed, it has been reported that, in low- and middle-income countries across Asia and sub-Saharan Africa, stunting, wasting, and thinness in women have decreased, while overweight and obesity have increased in the majority of age groups (29). Industrialization and urbanization in Asia have made low-nutrient foods and beverages easily accessible and affordable while promoting a sedentary lifestyle, thereby contributing to the DBM.

In addition, the malnourished obese patients tended to have more severe disease and the highest prevalence of atrial fibrillation (Table 2), which was consistent with previous studies. Obese populations exhibit a higher incidence, greater severity, and increased progression of atrial fibrillation compared with normal-weight individuals. Weight change appears to be associated with alterations in arrhythmia profile, highlighting obesity as a potential target for intervention (30). In addition, malnutrition also plays an important role in the development of atrial fibrillation (31), is associated with increased 1-year mortality in the elderly (32), and contributes to atrial fibrillation recurrence post-ablation (33). The potential relationship between atrial fibrillation, obesity, and malnutrition warrants further investigation to optimize the treatment of atrial fibrillation in obese individuals with DBM.

### Less favorable stroke outcomes in malnourished obese individuals receiving IVT

In our study, the malnourished obese AIS patients treated with IVT were at a significantly higher risk of poor outcomes and END compared with all other subgroups (Table 3; Supplementary Tables S5, S6; Figure 3; Supplementary Figure S1), consistent with findings from another cohort study among asymptomatic community-based adults in Taiwan, in which the malnourished obese group had the highest multivariable-adjusted risk of the composite outcome, including heart failure hospitalization and all-cause mortality (34). In contrast, our previous study found that obesity and malnutrition were correlated with the clinical prognosis of AIS patients who did not receive IVT or endovascular thrombectomy therapy at 3 months. The study showed that malnourished patients had the poorest prognosis, and the obesity paradox was observed, with the worst functional outcomes (34.4%) and the highest all-cause mortality (6.9%) occurring in malnourished non-obese participants (7). A similar trend was observed in patients with acute myocardial infarction (AMI), with the least favorable survival observed in the malnourished non-obese group, followed by the malnourished obese, nourished non-obese, and nourished obese groups (35).

The discrepancy related to the malnourished obese phenotype between AMI/AIS and heart failure may be associated with the acuity of the disease, as the cardiac insult in heart failure is a relatively more chronic condition compared to AMI/AIS (35). In heart failure, adipose tissue, such as that involved in cardiac steatosis, and its related comorbidities and metabolic dysregulation could promote a chronic pro-inflammatory process, accelerating cardiovascular dysfunction and structural remodeling of the myocardium over time.

Additionally, the obesity paradox, which refers to a favorable prognosis in individuals with obesity compared with their non-obese counterparts, has been reported in AIS patients (36). From a mechanistic perspective, the obesity paradox might be explained by inflammatory preconditioning in obese patients, who frequently present with chronic, low-grade inflammation and exhibit a reduced inflammatory response during acute illness. In addition, a higher BMI is typically associated with better nutritional status, and obese individuals may benefit from greater energy reserves during the initial catabolic phase of acute insults, owing to enhanced energy storage in adipose tissue (37–39). However, the obesity paradox has not been observed in patients treated with IVT, and some studies have suggested higher mortality rates among obese AIS patients after IVT. This discrepancy in prognostic outcomes warrants further investigation in large-scale prospective studies, as obese patients may be prone to more in-hospital complications such as venous thromboembolism, and the clot-dissolving effect of alteplase might be constrained by plasminogen activator inhibitor-1 overexpressed in adipose tissue (36). Notably, compared with well-nourished participants, the malnourished participants had worse clinical outcomes, particularly among individuals with severe malnutrition, irrespective of body mass category across all those studies.

### Study limitations

First, this single-center Chinese cohort inevitably suffers from limited external validity. Our conclusions cannot be directly generalized to other regions of China or to various ethnic groups worldwide, and cross-regional replicated trials are therefore essential. Second, only the CONUT score was used to evaluate patients’ nutritional status in this analysis, whereas nutritional status should be comprehensively evaluated by trained physicians using gold-standard measurements, such as the Global Leadership Initiative on Malnutrition (GLIM) criteria. The influence of the DBM on AIS patients receiving IVT within the context of comprehensive definitions, such as GLIM, requires further investigation for accurate interpretation. Third, wide confidence intervals were observed in several multivariable analyses, which were primarily attributed to the relatively limited sample size and a small number of clinical events. This may weaken the reliability of the observed associations, and large studies are required to verify our findings. In addition, given the relatively low number of outcome events, the adjusted estimates should be interpreted cautiously because of potential model overfitting. Finally, although BMI is a practical measure, it has limitations in distinguishing between different adipose tissue compartments, including visceral, subcutaneous, intramuscular, and intermuscular fat. Therefore, future multicenter, large-sample studies using other obesity indicators, such as waist circumference, waist-to-hip ratio, and visceral obesity, are urgently needed to validate our results.

## Conclusion

In our study, more than half of the AIS patients treated with IVT were malnourished, including those who were overweight or obese. Compared with well-nourished individuals, participants with malnutrition exhibited a poorer prognosis. Furthermore, moderate-to-severe malnutrition independently elevated the risks of poor outcomes, all-cause mortality, END, and HT during the follow-up period. Importantly, the malnourished obese group was associated with the worst clinical outcomes among the four subgroups, with these patients demonstrating the highest risk of poor outcomes and END, followed by the malnourished non-obese, nourished obese, and nourished non-obese individuals, respectively. Comprehensive screening of both nutritional status and body mass status is therefore recommended for AIS patients receiving IVT to guide risk stratification and optimize secondary preventive interventions.

## Data Availability

The original contributions presented in the study are included in the article/Supplementary material, further inquiries can be directed to the corresponding authors.

## Author’s note

All authors confirm that the following manuscript is a transparent and honest account of the reported research. This research is related to a previous study by the same authors titled “Prognosis of patients with coexisting obesity and malnutrition after ischemic stroke: A cohort study.” The previous study was performed on all patients with ischemic stroke, excluding those receiving IVT, while the current study focused solely on AIS patients treated with IVT. Both analyses used the identical prospective cohort methodology explained in the previously published article.

## Supplementary material

SUPPLEMENTARY FIGURE S1 Distribution of 3-month early neurological deterioration (*P* = 0.019) and hemorrhagic transformation (*P* = 0.045) across the four study subgroups.

## Glossary

IVT: intravenous thrombolysis
AIS: acute ischemic stroke
BMI: body mass index
DBM: double burden of malnutrition
CONUT: Controlling Nutritional Status
END: early neurological deterioration
CT: computed tomography
MRI: magnetic resonance imaging
mRS: modified Rankin Scale
GNRI: Geriatric Nutritional Risk Index
PNI: Prognostic Nutritional Index
SCR: serum creatinine
AST: aspartate aminotransferase
ALT: serum alanine transaminase
NIHSS: National Institutes of Health Stroke Scale
HT: hemorrhagic transformation
NRI: net reclassification improvement
IDI: integrated discrimination improvement
DNT: door-to-needle time
OR: odds ratio
AMI: acute myocardial infarction
GLIM: Global Leadership Initiative on Malnutrition
