# Supplementary material for: Impact of coexisting obesity and malnutrition on the clinical outcomes of acute ischemic stroke patients treated with intravenous thrombolysis: a cohort-based study
Source: Front Nutr. 2026 Jul 31;13:1864875. doi: 10.3389/fnut.2026.1864875 (PMC13474584; doi:10.3389/fnut.2026.1864875)
Supplement: Supplementary file 1 [file Data_Sheet_1.pdf]

Supplementary Material

Table S1. Classification of malnutrition according to 3 different scoring systems

Nutritional Indices		Risk of Malnutrition			
		Absent	Mild	Moderate	Severe
CONUT, points		0-1	2-4	5-8	9-12
Formula	Albumin, g/dL	≥3.50	3.00-3.49	2.50-2.99	＜2.50
	Total cholesterol, mg/dL	≥180	140-179	100-139	＜100
	Lymphocyte count, /mm^3	≥1,600	1,200-1,599	800-1,199	＜800
GNRI, points		＞98	92-98	82-92	＜82
Formula	1.489 × serum albumin [g/L] + 41.7 × present weight [kg]/ideal body weight [kg]				
PNI, points		＞38		35-38	＜35
Formula	serum albumin (g/L) + 5 × total lymphocyte (10^9/L)				

CONUT, Controlling Nutritional Status; GNRI, Geriatric Nutritional Risk Index; PNI, Prognostic Nutritional Index.

Table S2. Baseline characteristics between the enrolled patients and all Minhang Cohort patients

Risk Factors	Enrolled	Total	*P* value
**No. of patients**	366	6892	
Age (years)	68±12	70±13	0.001
Gender-Male, n (%)	239 (65.3%)	4379 (63.5%)	0.494
**Smoking, n (%)**	114 (31.1%)	1743 (25.3%)	**0.012**
**Drinking, n (%)**	52 (14.2%)	675 (9.8%)	0.006
**Medical history, n (%)**			
Hypertension	217 (59.3%)	4518 (65.6%)	0.014
Diabetes mellitus	108 (29.5%)	2130 (30.9%)	0.573
Stroke	43 (12.3%)	1188 (17.2%)	0.014
Hyperlipidemia	197 (53.8%)	3142 (45.6%)	0.002
Coronary artery disease	25 (6.8%)	681 (9.9%)	0.055
Atrial fibrillation	38 (10.4%)	623 (9.0%)	0.384
Admission NIHSS scores	6±5	5±6	<0.001
Admission mRS scores	0±0	0±1	0.039
Systolic pressure (mmHg)	150±21	144±21	<0.001
Diastolic pressure (mmHg)	85±13	82±12	<0.001
Time from onset to hospitalization (h）	1.6±0.9	22.8±33.6	<0.001
**Stroke subtype, n (%)**			0.109
LAA	199 (54.4%)	4107 (59.6%)	
CE	58 (15.8%)	933 (13.5%)	
SAO	103 (28.1%)	1679 (24.4%)	
Others	6 (1.6%)	172 (2.5%)	
**Laboratory tests**			

Hemoglobin (g/L)	138.3±17.2	133.9±18.7	<0.001
TG (mmol/L)	1.9±1.6	1.7±1.3	0.005
LDL (mmol/L)	3.0±1.0	2.8±1.0	<0.001
HDL (mmol/L)	1.2±0.3	1.1±0.3	<0.001
Creatinine (μmol/L)	79.4±22.1	85.5±50.0	0.019

NIHSS, National Institutes of Health Stroke Scale; mRS, modified Rankin Scale; LAA, large artery atherosclerosis; CE, cardioembolism; SAO, small-vessel occlusion; TG, triglyceride; TC, total cholesterol; LDL, Low-density lipoprotein; HDL, High-density lipoprotein.

Table S3. Severity of Malnutrition in the Study Cohort (n=366) based on 3 Different Scoring Systems.

	Absent	Mild	Moderate-Severe
CONUT	173 (47.3%)	174 (47.5%)	19 (5.2%)
GNRI	318 (86.9%)	31 (8.5%)	17 (4.6%)
PNI	362 (98.9%)	-	4 (1.1%)

CONUT, Controlling Nutritional Status; GNRI, Geriatric Nutritional Risk Index; PNI, Prognostic Nutritional Index.

Table S4. Comparative Analysis of the Discrimination of Each Malnutrition Score for Poor outcomes and All-Cause Mortality

Poor outcomes

Discrimination ability	CONUT		PNI		GNRI	
Sensitivity, %	69.3		45.5		53.4	
C-statistics	0.640		0.629		0.512	
95%CI	0.574-0.706		0.560-0.698		0.441-0.583	
	**CONUT vs. PNI**		**CONUT vs. GNRI**		**PNI vs. GNRI**	
**Comparison**	**Difference**	***P*-value**	**Difference**	***P*-value**	**Difference**	***P*-value**
C-statistics	0.011	0.709	0.117	0.005	0.128	<0.001
NRI	0.065	0.276	0.211	<0.001	0.146	0.008
IDI	0.009	0.365	0.051	<0.001	0.042	<0.001

All-Cause Mortality

Discrimination ability	CONUT		PNI		GNRI	
Sensitivity, %	75.0		43.2		66.7	
C-statistics	0.767		0.769		0.568	
95%CI	0.633-0.901		0.640-0.898		0.423-0.713	
	**CONUT vs. PNI**		**CONUT vs. GNRI**		**PNI vs. GNRI**	
**Comparison**	**Difference**	***P*-value**	**Difference**	***P*-value**	**Difference**	***P*-value**
C-statistics	0.002	0.945	0.199	0.029	0.201	0.038
NRI	0.071	0.521	0.459	<0.001	0.388	0.007
IDI	0.017	0.446	0.102	0.002	0.084	<0.001

Table S5. Clinical Outcomes of the Study Population

	Overall (N=366)	Nourished non-obese	Malnourished non-obese	Nourished obese	Malnourished obese	*P*-value

		(N=147)	(N=164)	(N=26)	(N=29)	
Poor outcomes	88 (24.0%)	22 (14.9%)	49 (29.8%)	5 (19.2%)	12 (41.3%)	0.002
All-cause mortality	12 (3.2%)	1 (0.6%)	8 (4.8%)	0 (0.0%)	3 (10.3%)	0.019
END	15 (4.1%)	2 (1.4%)	8 (4.9%)	1 (3.8%)	4 (13.8%)	0.019
HT	41 (11.2%)	10 (6.8%)	25 (15.2%)	1 (3.8%)	5 (17.2%)	0.045

END, early neurological deterioration, HT, hemorrhagic transformation.

Table S6. Adjusted Early Neurological Deterioration and Hemorrhagic Transformation based on Nutrition and Obesity Status

	Case (%)	OR (95% CI)	*P*-value
**END**	15 (4.1%)		
Nourished non-obese	2 (1.4%)	reference	
Malnourished non-obese	8 (4.9%)	6.564 (1.179-36.529)	0.032
Nourished obese	1 (3.8%)	4.745 (0.317-71.024)	0.259
Malnourished obese	4 (13.8%)	19.158 (2.324-157.935)	0.006
**HT**	41 (11.2%)		
Nourished non-obese	10 (6.8%)	reference	
Malnourished non-obese	25 (15.2%)	1.966 (0.874-4.429)	0.103
Nourished obese	1 (3.8%)	0.673 (0.078-5.788)	0.719
Malnourished obese	5 (17.2%)	1.377 (0.363-5.221)	0.638

Adjusted for age, admission NIHSS score, male sex, DNT, smoking, drinking, stroke subtype, diabetes, prior stroke, hypertension, atrial fibrillation, coronary heart disease, and statin use.. END, early neurological deterioration, HT, hemorrhagic transformation, NIHSS, National Institutes of Health Stroke Scale; DNT, door-to-needle time.

Table S7. Adjusted clinical outcomes based on malnutrition severity

	Case (%)	OR (95% CI)	*P*-value
**Poor outcomes**	88 (24.0%)		
None	27 (7.4%)	Reference	
Mild malnutrition	50 (13.7%)	1.786 (1.011-3.154)	0.046
Moderate-to-severe malnutrition	11 (3.0%)	5.282 (1.723-16.191)	0.004
**All-cause mortality**	12 (3.2%)		
None	1 (0.3%)	Reference	
Mild malnutrition	8 (2.2%)	6.240 (0.696-55.964)	0.102
Moderate-to-severe malnutrition	3 (0.8%)	31.729 (2.001-503.139)	0.014
**END**	15 (4.1%)		
None	3 (0.8%)	Reference	
Mild malnutrition	10 (2.7%)	5.208 (1.225-22.148)	0.025
Moderate-to-severe malnutrition	2 (0.5%)	17.368 (1.661-181.572)	0.017
**HT**	41 (11.2%)		
None	11 (3.0%)	Reference	
Mild malnutrition	23 (6.3%)	1.720 (0.774-3.820)	0.183
Moderate-to-severe malnutrition	7 (1.9%)	6.080 (1.684-21.948)	0.006

Adjusted for age, admission NIHSS score, male sex, DNT, smoking, drinking, stroke subtype, diabetes, prior stroke, hypertension, atrial fibrillation, coronary heart disease, and statin use. END, early neurological deterioration, HT, hemorrhagic transformation, NIHSS, National Institutes of Health Stroke Scale; DNT, door-to-needle time.
