# Supplementary material for: Impact of coexisting obesity and malnutrition on the clinical outcomes of acute ischemic stroke patients treated with intravenous thrombolysis: a cohort-based study
Source: Front Nutr. 2026 Jul 31;13:1864875. doi: 10.3389/fnut.2026.1864875 (PMC13474584; doi:10.3389/fnut.2026.1864875)
Supplement: Supplementary file 2 [file Table_1.docx]

Supplementary Material

**Table S1.** Classification of malnutrition according to 3 different scoring systems

| **Nutritional Indices** |  | **Risk of Malnutrition** | | | |
| --- | --- | --- | --- | --- | --- |
|  |  | **Absent** | **Mild** | **Moderate** | **Severe** |
| CONUT, points |  | 0-1 | 2-4 | 5-8 | 9-12 |
| Formula | Albumin, g/dL | ≥3.50 | 3.00-3.49 | 2.50-2.99 | ＜2.50 |
|  | Total cholesterol, mg/dL | ≥180 | 140-179 | 100-139 | ＜100 |
|  | Lymphocyte count, /mm^3^ | ≥1,600 | 1,200-1,599 | 800-1,199 | ＜800 |
| GNRI, points |  | ＞98 | 92-98 | 82-92 | ＜82 |
| Formula | 1.489 × serum albumin [g/L] + 41.7×present weight [kg]/ideal body weight [kg] | | | | |
| PNI, points |  | ＞38 |  | 35-38 | ＜35 |
| Formula | serum albumin (g/L) + 5×total lymphocyte (10^9^/L) | | | | |

CONUT, Controlling Nutritional Status; GNRI, Geriatric Nutritional Risk Index; PNI, Prognostic Nutritional Index.

**Table S2.** Baseline characteristics between the enrolled patients and all Minhang Cohort patients

| **Risk Factors** | **Enrolled** | **Total** | ***P* value** |
| --- | --- | --- | --- |
| **No. of patients** | 366 | 6892 |  |
| Age (years) | 68±12 | 70±13 | 0.001 |
| Gender-Male, n (%) | 239 (65.3%) | 4379 (63.5%) | 0.494 |
| **Smoking, n (%)** | 114 (31.1%) | 1743 (25.3%) | **0.012** |
| **Drinking, n (%)** | 52 (14.2%) | 675 (9.8%) | 0.006 |
| **Medical history, n (%)** |  |  |  |
| Hypertension | 217 (59.3%) | 4518 (65.6%) | 0.014 |
| Diabetes mellitus | 108 (29.5%) | 2130 (30.9%) | 0.573 |
| Stroke | 43 (12.3%) | 1188 (17.2%) | 0.014 |
| Hyperlipidemia | 197 (53.8%) | 3142 (45.6%) | 0.002 |
| Coronary artery disease | 25 (6.8%) | 681 (9.9%) | 0.055 |
| Atrial fibrillation | 38 (10.4%) | 623 (9.0%) | 0.384 |
| Admission NIHSS scores | 6±5 | 5±6 | <0.001 |
| Admission mRS scores | 0±0 | 0±1 | 0.039 |
| Systolic pressure (mmHg) | 150±21 | 144±21 | <0.001 |
| Diastolic pressure (mmHg) | 85±13 | 82±12 | <0.001 |
| Time from onset to hospitalization (h） | 1.6±0.9 | 22.8±33.6 | <0.001 |
| **Stroke subtype, n (%)** |  |  | 0.109 |
| LAA | 199 (54.4%) | 4107 (59.6%) |  |
| CE | 58 (15.8%) | 933 (13.5%) |  |
| SAO | 103 (28.1%) | 1679 (24.4%) |  |
| Others | 6 (1.6%) | 172 (2.5%) |  |
| **Laboratory tests** |  |  |  |
| Hemoglobin (g/L) | 138.3±17.2 | 133.9±18.7 | <0.001 |
| TG (mmol/L) | 1.9±1.6 | 1.7±1.3 | 0.005 |
| LDL (mmol/L) | 3.0±1.0 | 2.8±1.0 | <0.001 |
| HDL (mmol/L) | 1.2±0.3 | 1.1±0.3 | <0.001 |
| Creatinine (μmol/L) | 79.4±22.1 | 85.5±50.0 | 0.019 |

NIHSS, National Institutes of Health Stroke Scale; mRS, modified Rankin Scale; LAA, large artery atherosclerosis; CE, cardioembolism; SAO, small-vessel occlusion; TG, triglyceride; TC, total cholesterol; LDL, Low-density lipoprotein; HDL, High-density lipoprotein.

**Table S3.** Severity of Malnutrition in the Study Cohort (n=366) based on 3 Different Scoring Systems.

|  | **Absent** | **Mild** | **Moderate-Severe** |
| --- | --- | --- | --- |
| CONUT | 173 (47.3%) | 174 (47.5%) | 19 (5.2%) |
| GNRI | 318 (86.9%) | 31 (8.5%) | 17 (4.6%) |
| PNI | 362 (98.9%) | - | 4 (1.1%) |

CONUT, Controlling Nutritional Status; GNRI, Geriatric Nutritional Risk Index; PNI, Prognostic Nutritional Index.

**Table S4.** Comparative Analysis of the Discrimination of Each Malnutrition Score for Poor outcomes and All-Cause Mortality

| **Poor outcomes** | | | |
| --- | --- | --- | --- |
| **Discrimination ability** | **CONUT** | **PNI** | **GNRI** |
| Sensitivity, % | 69.3 | 45.5 | 53.4 |
| C-statistics | 0.640 | 0.629 | 0.512 |
| 95%CI | 0.574-0.706 | 0.560-0.698 | 0.441-0.583 |
|  | **CONUT vs. PNI** | **CONUT vs. GNRI** | **PNI vs. GNRI** |
| **Comparison** | **Difference *P*-value** | **Difference *P*-value** | **Difference *P*-value** |
| C-statistics | 0.011 0.709 | 0.117 0.005 | 0.128 <0.001 |
| NRI | 0.065 0.276 | 0.211 <0.001 | 0.146 0.008 |
| IDI | 0.009 0.365 | 0.051 <0.001 | 0.042 <0.001 |
| **All-Cause Mortality** | | | |
| **Discrimination ability** | **CONUT** | **PNI** | **GNRI** |
| Sensitivity, % | 75.0 | 43.2 | 66.7 |
| C-statistics | 0.767 | 0.769 | 0.568 |
| 95%CI | 0.633-0.901 | 0.640-0.898 | 0.423-0.713 |
|  | **CONUT vs. PNI** | **CONUT vs. GNRI** | **PNI vs. GNRI** |
| **Comparison** | **Difference *P*-value** | **Difference *P*-value** | **Difference *P*-value** |
| C-statistics | 0.002 0.945 | 0.199 0.029 | 0.201 0.038 |
| NRI | 0.071 0.521 | 0.459 <0.001 | 0.388 0.007 |
| IDI | 0.017 0.446 | 0.102 0.002 | 0.084 <0.001 |

**Table S5.** Clinical Outcomes of the Study Population

|  | **Overall**  **(N=366)** | **Nourished non-obese**  **(N=147)** | **Malnourished**  **non-obese**  **(N=164)** | **Nourished obese**  **(N=26)** | **Malnourished**  **obese**  **(N=29)** | ***P-*value** |
| --- | --- | --- | --- | --- | --- | --- |
| Poor outcomes | 88 (24.0%) | 22 (14.9%) | 49 (29.8%) | 5 (19.2%) | 12 (41.3%) | 0.002 |
| All-cause mortality | 12 (3.2%) | 1 (0.6%) | 8 (4.8%) | 0 (0.0%) | 3 (10.3%) | 0.019 |
| END | 15 (4.1%) | 2 (1.4%) | 8 (4.9%) | 1 (3.8%) | 4 (13.8%) | 0.019 |
| HT | 41 (11.2%) | 10 (6.8%) | 25 (15.2%) | 1 (3.8%) | 5 (17.2%) | 0.045 |

END, early neurological deterioration, HT, hemorrhagic transformation.

**Table S6.** Adjusted Early Neurological Deterioration and Hemorrhagic Transformation based on Nutrition and Obesity Status

|  | **Case (%)** | **OR (95% CI)** | ***P*-value** |
| --- | --- | --- | --- |
| **END** | 15 (4.1%) |  |  |
| Nourished non-obese | 2 (1.4%) | reference |  |
| Malnourished non-obese | 8 (4.9%) | 6.564 (1.179-36.529) | 0.032 |
| Nourished obese | 1 (3.8%) | 4.745 (0.317-71.024) | 0.259 |
| Malnourished obese | 4 (13.8%) | 19.158 (2.324-157.935) | 0.006 |
| **HT** | 41 (11.2%) |  |  |
| Nourished non-obese | 10 (6.8%) | reference |  |
| Malnourished non-obese | 25 (15.2%) | 1.966 (0.874-4.429) | 0.103 |
| Nourished obese | 1 (3.8%) | 0.673 (0.078-5.788) | 0.719 |
| Malnourished obese | 5 (17.2%) | 1.377 (0.363-5.221) | 0.638 |

Adjusted for age, admission NIHSS score, male sex, DNT, smoking, drinking, stroke subtype, diabetes, prior stroke, hypertension, atrial fibrillation, coronary heart disease, and statin use.. END, early neurological deterioration, HT, hemorrhagic transformation, NIHSS, National Institutes of Health Stroke Scale; DNT, door-to-needle time.

**Table S7.** Adjusted clinical outcomes based on malnutrition severity

|  | **Case (%)** | | **OR (95% CI)** | ***P*-value** |
| --- | --- | --- | --- | --- |
| **Poor outcomes** | 88 (24.0%) | |  |  |
| None | 27 (7.4%) | | Reference |  |
| Mild malnutrition | 50 (13.7%) | | 1.786 (1.011-3.154) | 0.046 |
| Moderate-to-severe malnutrition | 11 (3.0%) | | 5.282 (1.723-16.191) | 0.004 |
| **All-cause mortality** | 12 (3.2%) | |  |  |
| None | | 1 (0.3%) | Reference |  |
| Mild malnutrition | | 8 (2.2%) | 6.240 (0.696-55.964) | 0.102 |
| Moderate-to-severe malnutrition | | 3 (0.8%) | 31.729 (2.001-503.139) | 0.014 |

| **END** | 15 (4.1%) |  |  |
| --- | --- | --- | --- |
| None | 3 (0.8%) | Reference |  |
| Mild malnutrition | 10 (2.7%) | 5.208 (1.225-22.148) | 0.025 |
| Moderate-to-severe malnutrition | 2 (0.5%) | 17.368 (1.661-181.572) | 0.017 |
| **HT** | 41 (11.2%) |  |  |
| None | 11 (3.0%) | Reference |  |
| Mild malnutrition | 23 (6.3%) | 1.720 (0.774-3.820) | 0.183 |
| Moderate-to-severe malnutrition | 7 (1.9%) | 6.080 (1.684-21.948) | 0.006 |

Adjusted for age, admission NIHSS score, male sex, DNT, smoking, drinking, stroke subtype, diabetes, prior stroke, hypertension, atrial fibrillation, coronary heart disease, and statin use. END, early neurological deterioration, HT, hemorrhagic transformation, NIHSS, National Institutes of Health Stroke Scale; DNT, door-to-needle time.
